# The association between physical activity and internet addiction in college students: a serial mediation analysis of interoceptive awareness and emotion regulation difficulties

**DOI:** 10.3389/fpsyg.2026.1888809

**Published:** 2026-10-06

**Authors:** Hemei Gong, Yang Yang

**Affiliations:** School of Physical Education, Chengdu Sport University, Chengdu, China

**Keywords:** college students, emotion regulation difficulties, internet addiction, interoceptive awareness, physical activity

## Abstract

**Purpose:**

This study examined the association between physical activity and internet addiction among college students and tested the indirect associations of interoceptive awareness and emotion regulation difficulties using a serial mediation model.

**Methods:**

A cross-sectional online survey was conducted among 677 Chinese college students. Standardized instruments were used to assess physical activity, interoceptive awareness, emotion regulation difficulties, and internet addiction. Descriptive statistics, correlation analyses, one-way analysis of variance (ANOVA), and serial mediation analyses were performed using SPSS 27.0 and Mplus 8.3. Indirect associations and their confidence intervals were estimated using 5,000 bootstrap resamples. Continuous and nonlinear PA specifications, alternative scoring, and measurement checks were added.

**Results:**

Participants were categorized into low-, moderate-, and high-level physical activity groups. High physical activity, relative to low physical activity, had a negative conditional direct association with internet addiction scores (β = −0.200, *p* = 0.002). The serial mediation model indicated that high versus low physical activity was positively associated with interoceptive awareness (β = 0.550, *p* < 0.001) and negatively associated with emotion regulation difficulties (β = −0.287, *p* < 0.001). Continuous PA was inversely associated with IA scores (B = −2.031 per 1,000 MET-min/week, 95% HC3 CI [−2.431, −1.632]). The categorical serial indirect-association intervals excluded zero under both bootstrap methods; the unadjusted continuous intervals did not clearly do so (B = −0.043; 95% BC CI [−0.103, 0.000]; percentile CI [−0.097, 0.003]).

**Conclusion:**

In this cross-sectional study of college students, physical activity was negatively associated with internet addiction, but indirect associations involving the intermediary scores were specification-dependent and limited by measurement weaknesses. While consistent with embodied cognition theory, these findings preclude causal inference regarding directionality, mechanisms, or intervention effects. The results should therefore be interpreted as observational and hypothesis-generating. Longitudinal and experimental studies are needed to clarify temporal and causal relationships.

## 1 Introduction

Internet use is integral to college life, but persistent and poorly controlled use may interfere with psychological, academic, and social functioning. In this study, internet addiction refers to the dimensional severity of impaired control over internet use and related functional interference (Young, 1998), rather than a formal clinical diagnosis. Higher internet addiction scores have been associated with depression, anxiety, sleep disturbance, and impaired academic and social functioning (Ho et al., 2014). A recent meta-analysis using the 20-item Internet Addiction Test estimated a pooled prevalence of 41.84% among university students, although estimates varied substantially across regions and cutoff values (Liu et al., 2025). These findings support the examination of potentially modifiable correlates of internet addiction among college students.

Physical activity is defined as any bodily movement produced by skeletal muscles that requires energy expenditure (World Health Organization, 2020). Evidence linking physical activity with mental health includes a review of reviews in children and adolescents (Biddle and Asare, 2011) and a large cross-sectional study associating exercise with lower mental health burden in US adults (Chekroud et al., 2018). Recent Chinese studies have reported negative associations between physical activity and internet addiction or profiles of problematic internet use among college students (Du et al., 2024; Luo et al., 2024; Peng et al., 2026). A meta-analysis of randomized controlled trials also found that exercise interventions reduced internet addiction scores among college students (Yan et al., 2025). However, intervention evidence does not explain the psychological processes associated with habitual physical activity. Recent mediation studies have examined self-esteem, self-control, loneliness, rumination, mindfulness, and distress (Du et al., 2024; Xu and Tang, 2024; Wang et al., 2025; Gür and Gür, 2025). However, few studies have examined whether interoceptive awareness and emotion regulation difficulties jointly account for the association between physical activity and internet addiction.

Embodied cognition theory provides a framework for examining these associations. The theory proposes that cognition and emotion are shaped by interactions among the body, the brain, and the environment (Varela et al., 1991). During physical activity, individuals often attend to breathing, heart rate, muscle tension, fatigue, and recovery. These experiences may shape how they perceive and use bodily information. Interoceptive awareness is defined here as a multidimensional self-reported construct that reflects how individuals notice, interpret, and respond to internal bodily sensations (Mehling et al., 2018). A recent randomized trial found that body awareness psychotherapy reduced internet addiction among university students, with emotion regulation statistically mediating the intervention effect (Fallah et al., 2025). However, the study did not examine habitual physical activity or test interoceptive awareness and emotion regulation difficulties as sequential mediators. Among Chinese college students, physical activity was positively associated with several dimensions of interoceptive awareness (Sun et al., 2024). A recent systematic review similarly reported generally positive associations between exercise and interoceptive domains, although the available evidence was heterogeneous and subject to methodological limitations (Mulder et al., 2025). Evidence concerning internet-related problems is more limited. Di Carlo et al. (2024) found that people at risk of problematic internet use reported lower scores on selected dimensions of interoceptive awareness, while Simkute et al. (2025) reported that greater problematic internet use was associated with lower Not-Distracting and Trusting scores. Complementary evidence comes from a small randomized trial in which body awareness psychotherapy reduced internet addiction among university students, with emotion regulation statistically mediating the intervention effect (Fallah et al., 2025). However, that study examined a structured psychological intervention rather than habitual physical activity and did not test interoceptive awareness and emotion regulation difficulties as sequential mediators. While these findings highlight interoceptive awareness—and its specific dimensions—as a crucial link between physical activity and internet addiction, the data preclude confirming the model’s proposed temporal causality.

Emotion regulation difficulties may provide a second link. This construct refers to problems in understanding and accepting emotions, maintaining goal-directed behavior, controlling impulses, and accessing effective regulation strategies (Gratz and Roemer, 2004). A recent single-group study reported lower emotion regulation difficulties after a short physical activity program, but the absence of a control group limits causal interpretation (Popescu et al., 2025). Evidence linking emotion regulation difficulties to internet addiction is more direct. A one-year prospective study found that impulse-control difficulties predicted the onset of internet addiction in male college students, although this pattern was not uniform across sex or all dimensions (Tsai et al., 2020). A recent pilot study also found a positive cross-sectional correlation between the two variables among university students (Hainagiu and Neagu, 2025). Research in adolescents has also identified associations between specific emotion-regulation strategies and internet addiction (Yildiz, 2017). The compensatory internet use model proposes that students who struggle to manage distress may use the internet for short-term relief or avoidance (Kardefelt-Winther, 2014), and repeated reliance on this response may coincide with poorer control over internet use.

Interoceptive awareness and emotion regulation difficulties are conceptually connected. Bodily changes provide information that can help a person identify an emotional state and select a response. The somatic marker hypothesis places such body-based information within judgment and behavioral regulation (Damasio, 1996). Several adaptive dimensions of interoceptive awareness are also associated with lower levels of emotion regulation difficulties, although the associations differ across dimensions (Desdentado et al., 2023). A recent randomized study found lower internet addiction symptoms after body awareness psychotherapy, with a significant indirect effect through changes in emotion regulation difficulties (Fallah et al., 2025). That study did not assess habitual physical activity or the serial pathway examined here. The literature reviewed for this study did not identify research that tested interoceptive awareness and emotion regulation difficulties simultaneously as sequential mediators between physical activity and internet addiction. Examining this integrated model constitutes the main contribution of the present study.

Nevertheless, the proposed sequence is not the only plausible direction. Students with lower internet addiction may have more time, energy, or motivation for physical activity. Internet addiction may also displace active behavior through prolonged screen time. Emotion regulation difficulties may reduce physical activity, while internet use may serve as an alternative coping response. Thus, reciprocal and reverse associations remain theoretically plausible. The cross-sectional design of the present study cannot distinguish among these competing directions.

Accordingly, this study examines the association between physical activity and internet addiction among Chinese college students. It further examines indirect statistical associations involving interoceptive awareness and emotion regulation difficulties, both separately and sequentially. The hypotheses are as follows:

H1: Physical activity is negatively associated with internet addiction.

H2: Interoceptive awareness showed a statistically significant indirect association between physical activity and internet addiction.

H3: Emotion regulation difficulties statistically mediate the association between physical activity and internet addiction.

H4: A serial indirect association is expected between physical activity and internet addiction through interoceptive awareness and emotion regulation difficulties, such that higher physical activity is associated with higher interoceptive awareness, lower emotion regulation difficulties, and lower internet addiction.

## 2 Methodology

### 2.1 Participants

Participants were full-time university students recruited across Sichuan Province, China utilizing the Wenjuanxing online platform. To ensure data integrity, the system restricted access to a single response per IP address and device. An *a priori* power analysis (G*Power 3.1; two-tailed test, α = 0.05, 1 − β = 0.80) established a minimum required sample of 109 (Faul et al., 2009). The survey was distributed via closed student networks, yielding 700 initial responses. We excluded 23 surveys based on objective criteria: failed attention checks, speeding (completion time < [300] s), or straight-lining patterns. The final sample comprised 677 valid responses (96.7% retention), fully satisfying statistical power requirements. The sample comprised 677 students, including 344 men (50.8%) and 333 women (49.2%). Most participants were 18–22 years old (*n* = 537, 79.3%), and both undergraduate and graduate students were represented. Daily internet-use time was recorded in five ordered response categories (range = 1–5; M = 2.89, SD = 1.065). The primary purposes of internet use were social networking (76.2%), short videos or live streaming (66.0%), and study or research (62.3%). Secondary purposes included gaming (49.3%), instant messaging (46.5%), and online shopping (38.7%). Gender, age group, and daily internet-use time were examined as candidate covariates in a sensitivity analysis; their coding and analytic role are described in the Statistical Analysis section.

### 2.2 Procedure

This study employed a cross-sectional design, administering questionnaires to assess all variables at a single time point. Prior to the main survey, the research team standardized the instrument’s content, instructions, and administration protocols. A subsequent pilot test (N = 100, yielding 92 valid responses) was conducted to evaluate item clarity and procedural feasibility. Insights drawn from this pilot phase informed minor refinements to item wording and presentation format, ensuring optimal comprehension. During the formal data collection, participants were recruited voluntarily via online platforms using survey links and QR codes. To uphold ethical standards, access to the questionnaire was strictly contingent upon participants reading and providing digital informed consent. The finalized instrument captured demographic data alongside validated measures of physical activity, interoceptive awareness, emotion regulation difficulties, and internet addiction. Emphasizing anonymity and confidentiality, the entire process adhered strictly to the ethical guidelines approved by the Institutional Review Board of Chengdu Sport University (Approval Number: 2025146).

### 2.3 Measures

#### 2.3.1 International physical activity questionnaire–short form (IPAQ-SF)

Physical activity was assessed using the International Physical Activity Questionnaire–Short Form (IPAQ-SF), which has shown acceptable reliability and validity in Chinese populations (Macfarlane et al., 2007). Participants reported the frequency and duration of walking, moderate-intensity activity, and vigorous-intensity activity during the previous 7 days. Following the IPAQ scoring protocol, activity lasting <10 min/day was coded as zero, durations >180 min/day were truncated to 180 min/day, and MET values of 3.3, 4.0, and 8.0 were assigned to walking, moderate, and vigorous activity, respectively. Total physical activity was calculated as MET-min/week by summing MET × minutes/day × days/week across activity intensities. Participants were classified as having low, moderate, or high physical activity according to standard IPAQ criteria combining frequency, duration, intensity, and accumulated MET-min/week (IPAQ Research Committee, 2005). These protocol-defined categories were not based on sample tertiles or outcome-derived cut points. Sensitivity analyses additionally modeled total MET-min/week continuously (per 1,000 MET-min/week) and nonlinearly (Supplementary Tables S1–S3, S12).

#### 2.3.2 Multidimensional assessment of interoceptive awareness, version 2 (MAIA-2)

Interoceptive awareness was assessed using the Multidimensional Assessment of Interoceptive Awareness, Version 2 (MAIA-2; Mehling et al., 2018). We employed the Chinese-language MAIA-2; the Chinese validation by Teng et al. (2022) supported a refined structure rather than the 37-item general score. The instrument contains 37 items rated from 0 (never) to 5 (always) across eight domains: Noticing, Not-Distracting, Not-Worrying, Attention Regulation, Emotional Awareness, Self-Regulation, Body Listening, and Trusting. The present study retained the original 37-item, eight-domain structure, whereas the Chinese validation by Teng et al. (2022) supported a refined 31-item, seven-factor structure. After reverse scoring was verified, the primary analysis used an exploratory composite derived from all 37 items by averaging the eight domain scores, with higher values indicating greater self-reported interoceptive awareness across the retained domains. The scale showed good internal consistency in the present sample (Cronbach’s α = 0.802).

#### 2.3.3 Difficulties in emotion regulation scale (DERS)

Emotion regulation difficulties were assessed using the 36-item Difficulties in Emotion Regulation Scale (DERS; Gratz and Roemer, 2004). The Chinese version has demonstrated satisfactory reliability and validity among Chinese adults (Li et al., 2018). Items are rated on a 5-point Likert scale from 1 (almost never) to 5 (almost always) and assess six domains: Nonacceptance of Emotional Responses, Difficulties Engaging in Goal-Directed Behavior, Impulse Control Difficulties, Lack of Emotional Awareness, Limited Access to Emotion Regulation Strategies, and Lack of Emotional Clarity. After reverse scoring the relevant items, responses were summed across all 36 items, with higher scores indicating greater emotion regulation difficulties. In the present sample, the total scale showed good internal consistency (Cronbach’s α = 0.827).

#### 2.3.4 Internet Addiction Test (IAT)

Internet addiction was assessed using Young’s Internet Addiction Test (IAT; Young, 1998). The IAT is a 20-item self-report measure designed to assess the frequency and duration of internet use and its impact on different aspects of daily life. Items are rated on a 5-point Likert scale ranging from 1 (almost never) to 5 (always). Total scores were calculated by summing all item responses and ranged from 20 to 100, with higher scores indicating greater internet addiction. The Chinese-language IAT has demonstrated satisfactory reliability and validity among Chinese student samples (Lu et al., 2022; Wei et al., 2025). In the present sample, the IA showed good internal consistency (Cronbach’s α = 0.896).

### 2.4 Statistical analysis

Statistical analyses were conducted using SPSS 27.0 and Mplus 8.3. SPSS was used for preliminary, group-comparison, and robustness checks. Descriptive statistics, Pearson correlations, and variance inflation factors (VIFs) were calculated first. Group differences across PA levels were tested using Welch’s ANOVA for IAw and conventional one-way ANOVAs for ERD and IA. Games–Howell *post hoc* tests were used for pairwise comparisons (Supplementary Tables S4, S5), whereas HC3 robust standard errors were used for regression models (Supplementary Table S2). Robustness was further examined by modelling PA categorically and continuously, including linear and restricted cubic spline (RCS) functions (Supplementary Table S2; Supplementary Figure S1). Regression diagnostics were conducted, and adjusted models controlled for gender (female vs. male), age group, and daily internet-use time (Supplementary Tables S2–S4; Supplementary Figure S2). In Mplus, confirmatory factor analyses (CFAs) and serial indirect-association models were conducted for the primary and sensitivity specifications. The three-factor CFA included eight IAw subscale scores, six ERD subscale scores, and four IA item parcels, excluding PA (Supplementary Table S6). CFA used maximum likelihood with robust standard errors (MLR), with fit assessed using χ^2^, CFI, TLI, SRMR, and RMSEA. Serial models used maximum likelihood, with PA as predictor, IAw and ERD as ordered intermediaries, and IA as outcome (Hayes, 2018). In the primary model, PA was dummy-coded with the low-activity group as reference. PA2 represented moderate versus low physical activity, and PA3 represented high versus low physical activity. Indirect associations were evaluated using 5,000 bootstrap samples. Primary decompositions reported BC and percentile 95% confidence intervals, with BC intervals treated as primary, together with model-based Wald *p* values (Supplementary Table S11). Intervals reaching zero at reported precision were not treated as clearly excluding zero. We reported standardized and unstandardized estimates, *p* values, bootstrap confidence intervals, and R^2^ values. These decompositions do not establish temporal ordering, causal mediation, or causal mechanisms. Sensitivity analyses examined categorical, linear, and restricted cubic spline PA specifications (Supplementary Tables S2, S10, S12). An additional serial model included gender, age group, and daily internet-use time as covariates predicting IAw, ERD, and IA (Supplementary Table S9). Primary analyses retained all IAw and ERD dimensions; *post hoc* analyses evaluated MAIA-31 and DERS-30 scoring. Further details, including reliability analyses and higher-order CFAs, are provided in Supplementary Table S12.

## 3 Results

### 3.1 Data completeness and analytic assumptions

All 677 participants had complete data for the variables used in the analyses; therefore, no imputation was required. Physical activity was analysed as a three-level categorical variable: low (*n* = 99, 14.6%), moderate (*n* = 195, 28.8%), and high (*n* = 383, 56.6%). For the primary categorical models, residual diagnostics did not indicate marked departures from model assumptions, and the largest VIF was 2.114 (Supplementary Table S4). The homogeneity-of-variance assumption was not supported for interoceptive awareness, Levene’s *F*(2, 674) = 32.365, *p* < 0.001, but was supported for emotion regulation difficulties, *F*(2, 674) = 1.824, *p* = 0.162, and internet addiction, *F*(2, 674) = 1.280, *p* = 0.279. Welch’s ANOVA was therefore used for interoceptive awareness, *F*(2, 302.563) = 121.789, *p* < 0.001, whereas conventional one-way ANOVAs were used for the other outcomes. Games–Howell tests were used for pairwise comparisons (Supplementary Tables S4, S5). In the continuous-PA sensitivity specification, heteroskedasticity was identified in the interoceptive-awareness and emotion-regulation-difficulties equations, and nonlinearity was identified in the internet-addiction equation. HC3 confidence intervals and restricted cubic-spline sensitivity analyses were therefore reported (Supplementary Table S3, and Supplementary Figures S1–S2).

### 3.2 Measurement model and common method bias

Common method bias (CMB) was addressed through procedural remedies and assessed using an unrotated principal component analysis of all original PA, IAw, ERD, and IA items. The item matrix was factorable (KMO = 0.833; Bartlett’s χ^2^(4,950) = 24170.773, *p* < 0.001). Although 27 components had eigenvalues > 1, the first accounted for 10.376% of the total variance, below the commonly used 40% criterion (Podsakoff et al., 2003). Thus, no dominant single method factor was evident; however, residual common method variance cannot be excluded.

A three-factor confirmatory factor analysis (CFA) was estimated using MLR to evaluate the aggregate measurement model. The model included eight IAw-2 subscale scores, six ERD subscale scores, and four balanced IA item parcels; PA was excluded because it was an observed categorical variable. Global model fit was acceptable, χ^2^(132) = 204.559, *p* < 0.001, CFI = 0.972, TLI = 0.967, RMSEA = 0.028, 90% CI [0.021, 0.036], and SRMR = 0.042. However, several standardised factor loadings were weak, including those for IAw Noticing (0.140), Not-Distracting (0.142), and Not-Worrying (0.186), and for ERD Awareness (0.086) and Strategies (0.025). In contrast, IA parcel loadings ranged from 0.820 to 0.840 (Supplementary Table S6). Total-score reliability was adequate for 37-item IAw (α = 0.802), 36-item ERD (α = 0.827), and 20-item IA (α = 0.896). However, reliability was low for the ERD Nonacceptance subscale (α = 0.395, 95% bootstrap CI [0.308, 0.470]) and Awareness subscale (α = 0.571, 95% bootstrap CI [0.509, 0.623]). Alternative MAIA-31 and DERS-30 scoring specifications yielded α values of 0.806 and 0.851, respectively (Supplementary Table S7). In higher-order ERD CFAs estimated using WLSMV, the standardised loadings for Awareness (0.138) and Strategies (0.011) were weak; the Strategies loading remained weak (0.012) after Awareness was omitted. Global fit was acceptable for the 36-item model (CFI = 0.955, TLI = 0.952, RMSEA = 0.050, SRMR = 0.054) and the 30-item model (CFI = 0.962, TLI = 0.958, RMSEA = 0.055, SRMR = 0.056; Supplementary Table S8). These local measurement limitations should be considered when interpreting the composite intermediary scores.

### 3.3 Descriptive statistics and correlations

To examine the associations among the study variables, Table 1 presents the descriptive statistics and Pearson correlations for interoceptive awareness, emotion regulation difficulties, and internet addiction, all treated as continuous variables. Physical activity was not included in this correlation matrix because it was represented by three categories. Interoceptive awareness was negatively correlated with emotion regulation difficulties (*r* = −0.353, *p* < 0.001) and internet addiction (*r* = −0.227, *p* < 0.001), whereas emotion regulation difficulties were positively correlated with internet addiction (*r* = 0.216, *p* < 0.001). These results were consistent with the study hypotheses.

**Table 1:** Descriptive statistics and correlation analysis of the main variables (*N* = 677).

Variable	M	SD	IAw	ERD	IA
IAw	2.299	0.358	—		
ERD	91.415	8.614	−0.353***	—	
IA	43.750	11.928	−0.227***	0.216***	—

IAw, interoceptive awareness; ERD, emotion regulation difficulties; IA, Internet Addiction; M, mean; SD, standard deviation; *p < 0.05; **p < 0.01; ***p < 0.001.

### 3.4 Comparisons across physical-activity levels

Physical-activity groups differed on all three outcomes (Table 2). For interoceptive awareness, the conventional omnibus test was significant, *F*(2, 674) = 106.971, *p* < 0.001, with a descriptive η^2^ = 0.241. The HC3 omnibus test also indicated a significant group difference, *F*(2, 674) = 121.484, *p* < 0.001. Games–Howell comparisons showed that interoceptive awareness was higher in the moderate group than in the low group (MD = 0.069, 95% CI [0.002, 0.136], *p* = 0.041), and it was higher in the high group than in the moderate and low groups. Emotion regulation difficulties also differed by group, *F*(2, 674) = 54.655, *p* < 0.001, η^2^ = 0.140. The high group reported lower scores than the low and moderate groups (both *p* < 0.001), whereas the low and moderate groups did not differ significantly. Internet addiction showed the same group pattern, *F*(2, 674) = 22.726, *p* < 0.001, η^2^ = 0.063. The high group reported lower scores than the low and moderate groups (both *p* < 0.001), whereas the low–moderate contrast was not significant (*p* = 0.194; Supplementary Table S5).

**Table 2:** Psychological outcomes by physical-activity level (*N* = 677).

Variables	Low (*n* = 99) M (SD)	Moderate (*n* = 195) M (SD)	High (*n* = 383) M (SD)	*F*(2, 674)	*p*	η*^2^*	Games–Howell
IAw	2.054 (0.223)	2.123 (0.240)	2.452 (0.361)	106.971†	< 0.001	0.241	H > M > L
ERD	95.727 (7.386)	94.733 (7.723)	88.611 (8.288)	54.655	< 0.001	0.140	L = M > H
IA	48.680 (11.713)	46.235 (10.699)	41.210 (11.939)	22.726	< 0.001	0.063	L = M > H

Values are M (SD). L = low; M = moderate; H = high; η^2^ = eta squared. Groups joined by ≈ did not differ significantly. † For interoceptive awareness, Levene’s test indicated heterogeneity of variance; therefore, inference was based on HC3-robust estimates and Games–Howell pairwise comparisons. The HC3 omnibus test was significant, F(2, 674) = 121.484, *p* < 0.001, and both robust approaches supported H > M > L.

### 3.5 Primary serial indirect-association analysis

The observed-variable serial mediation model was saturated (df = 0); global fit indices were therefore uninformative. Using low physical activity as the reference group, PA2 and PA3 represented moderate and high activity, respectively, with interoceptive awareness (IAw) and emotion regulation difficulties (ERD) specified as ordered intermediary scores. Both contrasts were positively associated with IAw (PA2: β = 0.087, *p* = 0.014; PA3: β = 0.550, *p* < 0.001). IAw and PA3 were negatively associated with ERD (β = −0.224 and −0.287, respectively; both *p* < 0.001), whereas PA2 was not (β = −0.033, *p* = 0.489). In the final equation, ERD was positively associated with internet addiction (IA; β = 0.121, *p* = 0.005), whereas IAw was negatively associated with IA (β = −0.110, *p* = 0.009). Conditional direct associations were significant for PA3 (β = −0.200, *p* = 0.002), but not PA2 (β = −0.077, *p* = 0.147). The model explained 24.1, 17.7, and 9.0% of the variance in IAw, ERD, and IA, respectively (Table 3 and Figure 1).

**Table 3:** Regression paths in the serial statistical mediation model.

Outcome (R^2^)	Predictor	*b*	SE	β	*p*	95% BC bootstrap CI for b
IAw (R^2^ = 0.241)	PA2	0.069	0.028	0.087	0.014	[0.014, 0.123]
	PA3	0.398	0.029	0.550	< 0.001	[0.341, 0.455]
ERD (R^2^ = 0.177)	IAw	−5.374	0.956	−0.224	< 0.001	[−7.276, −3.495]
	PA2	−0.624	0.902	−0.033	0.489	[−2.301, 1.178]
	PA3	−4.979	0.947	−0.287	< 0.001	[−6.825, −3.097]
IA (R^2^ = 0.090)	ERD	0.168	0.060	0.121	0.005	[0.043, 0.279]
	IAw	−3.676	1.407	−0.110	0.009	[−6.374, −0.820]
	PA2	−2.021	1.393	−0.077	0.147	[−4.756, 0.781]
	PA3	−4.810	1.521	−0.200	0.002	[−7.872, −1.813]

PA2 = moderate versus low physical activity; PA3 = high versus low physical activity. Low physical activity was the reference group. b = unstandardized coefficient; SE = standard error; β = standardized coefficient; BC CI = bias-corrected bootstrap confidence interval based on 5,000 resamples. All *p* values are two-tailed. Because the model was saturated, R^2^ and individual path estimates, rather than global fit indices, were used to summarize the model.

**Figure 1:**
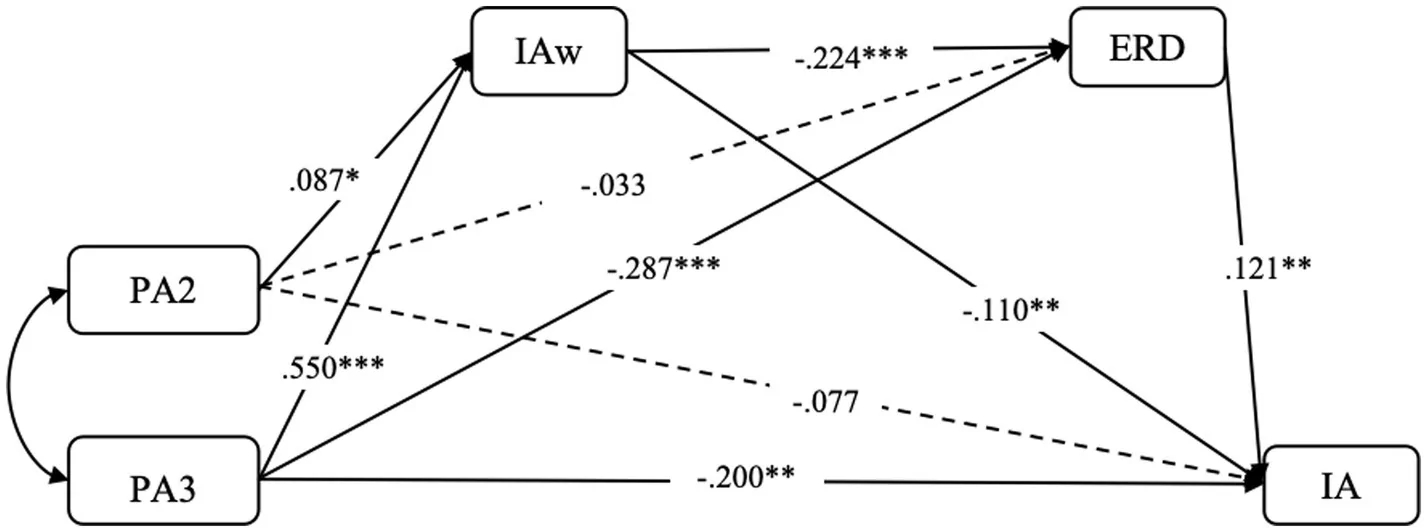
Standardized path coefficients for the serial mediation model. PA2, moderate physical activity; PA3, high physical activity (reference: low physical activity); IAw, interoceptive awareness; ERD, emotion regulation difficulties; IA, internet addiction. Solid and dashed single-headed arrows indicate significant and non-significant paths, respectively. *p < 0.05; **p < 0.01; ***p < 0.001. Paths do not establish temporal ordering or causality.

Table 4 presents the association decomposition. For PA2, neither the total nor the conditional direct association with internet addiction was significant. However, the total indirect association had a BC interval excluding zero (b = −0.420, 95% BC CI [−0.959, −0.011]) but a percentile interval including zero (Supplementary Table S11). The IAw-only and serial indirect intervals excluded zero under both bootstrap methods, whereas the ERD-only intervals included zero. Wald tests did not support the PA2 total indirect, IAw-only or serial estimates at 0.05. For PA3, the total, conditional direct, total indirect and all three specific indirect associations were statistically supported; the serial estimate was b = −0.358, 95% BC CI [−0.707, −0.113]. Across supplementary specifications, support for the serial indirect association was not uniform (Supplementary Table S12). Given the cross-sectional design, the indirect associations represent statistical decompositions of contemporaneous associations rather than evidence of causal mediation.

**Table 4:** Total and indirect association estimates in the serial statistical mediation model.

Contrast	Effect path	*b*	SE	β	*p*	95% BC bootstrap CI for b
PA2 (Moderate vs. Low)	Total effect	−2.441	1.404	−0.093	0.082	[−5.146, 0.422]
	Total indirect effect	−0.420	0.240	−0.016	0.080	[−0.959, −0.011]
	PA2 → IAw → IA	−0.253	0.150	−0.010	0.092	[−0.648, −0.036]
	PA2 → ERD → IA	−0.105	0.166	−0.004	0.529	[−0.497, 0.179]
	PA2 → IAw → ERD → IA	−0.062	0.038	−0.002	0.099	[−0.168, −0.010]
PA3 (High vs. Low)	Total effect	−7.465	1.326	−0.310	< 0.001	[−10.090, −4.836]
	Total indirect effect	−2.655	0.693	−0.110	< 0.001	[−4.016, −1.329]
	PA3 → IAw → IA	−1.462	0.575	−0.061	0.011	[−2.593, −0.331]
	PA3 → ERD → IA	−0.835	0.346	−0.035	0.016	[−1.635, −0.261]
	PA3 → IAw → ERD → IA	−0.358	0.151	−0.015	0.017	[−0.707, −0.113]

PA2 = moderate versus low physical activity; PA3 = high versus low physical activity; IAw = interoceptive awareness; ERD = emotion regulation difficulties; IA = internet addiction. Low physical activity was the reference group. b = unstandardized effect estimate; SE = standard error; β = standardized effect estimate. Standardized indirect effects were derived from the corresponding STDYX-standardized path coefficients. *p* values are two-tailed Wald-test p values based on maximum-likelihood standard errors. BC CI = 95% bias-corrected bootstrap confidence interval based on 5,000 resamples. For indirect associations, statistical inference was based primarily on whether the 95% BC CI excluded zero.

### 3.6 Sensitivity and robustness analyses

Sensitivity analyses examined continuous PA specifications, covariate adjustment (gender, age group, and daily internet-use time), alternative intermediary scoring, and a nonlinear PA specification. In regressions without intermediary scores, continuous PA was inversely associated with IA scores (B = −2.031 per 1,000 MET-min/week, 95% HC3 CI [−2.431, −1.632], *p* < 0.001). The covariate-adjusted estimate was similar (B = −1.963, 95% HC3 CI [−2.369, −1.556], *p* < 0.001; Supplementary Table S2). In the unadjusted continuous model, the serial indirect association was not clearly supported (B = −0.043, 95% BC CI [−0.103, 0.000]; 95% percentile CI [−0.097, 0.003]), as neither interval clearly excluded zero at the reported precision. The IAw-only, ERD-only, and total indirect intervals included zero under both bootstrap interval methods (Supplementary Table S11). After covariate adjustment, the continuous serial indirect estimate had a BC interval excluding zero (B = −0.043, 95% BC CI [−0.102, −0.003]; Supplementary Table S12). Continuous PA models using *post hoc* MAIA-31 and DERS-30 scores yielded serial estimates with BC intervals excluding zero (B = −0.038, 95% BC CI [−0.094, −0.003] and B = −0.048, 95% BC CI [−0.106, −0.006], respectively). The corresponding categorical serial BC intervals also excluded zero. The restricted cubic spline model yielded a serial indirect estimate of B = −0.148 for the 75th-versus-25th percentile PA contrast (95% BC CI [−0.340, −0.014]; Supplementary Table S12).

## 4 Discussion

Guided by embodied cognition theory, this cross-sectional study investigated the relationships among physical activity, interoceptive awareness, emotion regulation difficulties, and internet addiction in Chinese college students. Notably, students engaging in high levels of physical activity exhibited significantly lower internet addiction compared to their less active peers. This inverse relationship featured specification-dependent statistical indirect associations involving higher interoceptive awareness and lower emotion regulation difficulties; the inverse continuous PA–IA total association was more consistent than the indirect-association pattern. Although these observational patterns are compatible with our conceptual framework, the cross-sectional design precludes causal inferences. Furthermore, the model accounted for 9.0% of the variance in internet addiction, suggesting modest explanatory power and leaving substantial variance unexplained.

### 4.1 Association between physical activity and internet addiction

The study found that students in the high physical-activity group reported significantly lower internet addiction scores than those in the low-activity group, while the moderate- and low-activity groups did not differ significantly. This pattern is consistent with a recent meta-analysis showing that physical activity interventions reduced internet addiction among college students (Yan et al., 2025). It is also align with recent cross-sectional studies on Chinese and Tunisian university students, which reported an inverse association between physical activity and internet-related problems, while also identifying stress, anxiety, and depression as significant correlates (Luo et al., 2024; Jelleli et al., 2024). However, the present evidence does not establish that physical activity protects against internet addiction. The categorical group pattern also does not establish a linear dose–response association or a threshold effect. Continuous PA was inversely associated with IA scores before and after covariate adjustment, while exploratory spline results suggested curvature; these results do not establish a causal dose response or a discrete activity threshold. Reverse directionality is equally plausible. Students with lower internet addiction may have more time, energy, or motivation to participate in physical activity, whereas excessive internet use may displace active pursuits or coincide with fatigue and disrupted daily routines. Furthermore, unmeasured shared factors—such as self-control, peer context, and academic demands, may also contribute to both behaviors. The present design cannot distinguish among these explanations. Evidence from intervention research supports the plausibility of an effect of physical activity, but it does not convert the association observed in this study into a causal effect.

### 4.2 Indirect association involving interoceptive awareness

Our findings regarding interoceptive awareness align with an emerging, albeit limited, body of literature. Previous research has positively associated physical activity with multiple dimensions of interoceptive awareness among Chinese college students (Sun et al., 2024), while other studies have correlated specific facets—particularly Not-Distracting and Trusting—with problematic internet use (Di Carlo et al., 2024; Simkute et al., 2025). Together, these observational studies underscore the relevance of interoceptive awareness as a conceptual bridge between physical activity and internet addiction. This conceptual link is further reinforced by recent clinical evidence demonstrating that body awareness psychotherapy addresses internet addiction through the mediating roles of executive functions and emotion regulation (Fallah et al., 2025). Viewed through the lens of embodied cognition theory, which posits that cognition and emotion emerge from dynamic interactions among the body, brain, and environment (Varela et al., 1991)—heightened attention to breathing, heart rate, and muscle fatigue during exercise naturally corresponds to higher levels of interoceptive awareness. This sensory feedback is, in turn, conceptually tied to emotional processing and behavioral regulation (Critchley and Garfinkel, 2017; Price and Hooven, 2018). Despite these theoretical alignments, the cross-sectional nature of the present study precludes causal inferences. We cannot definitively assert that physical activity temporally precedes greater interoceptive awareness, nor that such awareness subsequently acts as a temporal precursor to lower internet addiction. Furthermore, lacking direct measurement of the anterior insula or other neural substrates, the underlying neurobiological mechanisms remain theoretically plausible but empirically untested within this study. However, the IAw-only indirect-association intervals did not exclude zero in the continuous PA specification. The weak Noticing, Not-Distracting, and Not-Worrying loadings limit interpretation of the exploratory domain-mean composite; the Chinese 31-item validation and item-level alpha do not validate a 37-item general score or this composite as a latent intermediary.

### 4.3 Indirect association involving emotion regulation difficulties

Emotion regulation difficulties were positively associated with internet addiction, whereas high physical activity was associated with lower levels of emotion regulation difficulties. A recent pilot study using the emotion regulation difficulties and internet addiction similarly reported a positive cross-sectional association between emotion regulation difficulties and internet addiction among university students (Hainagiu and Neagu, 2025). This observational pattern is compatible with the compensatory internet use model, which proposes that some individuals use the internet as a strategy to manage distress or avoid offline problems (Kardefelt-Winther, 2014). The updated I-PACE model likewise positions affective and executive processes at the center of problematic internet-use behaviors (Brand et al., 2019). Furthermore, a recent systematic review identified emotion-related processes as potential intermediaries in the associations between physical activity and mental health, although the available evidence varied in design and methodological quality (White et al., 2024). While these frameworks provide plausible psychological interpretations, the present cross-sectional data establish only contemporaneous associations. They cannot demonstrate that physical activity temporally precedes lower emotion regulation difficulties, nor that such difficulties act as a temporal precursor to internet addiction. Alternative directional relationships remain equally plausible: greater emotion regulation difficulties might simply co-occur with lower participation in physical activity, just as more severe internet addiction could coincide with physical inactivity or correspond to heightened emotional dysregulation. Accordingly, these psychological processes should be regarded as theory-informed associative possibilities rather than causal mechanisms demonstrated by the present study. The ERD-only indirect association was supported for the categorical high-versus-low contrast but not the unadjusted continuous specification. Weak Awareness and Strategies loadings, together with low Nonacceptance and Awareness reliability, limit interpretation of the total score as a coherent intermediary construct; excluding Awareness did not resolve the weak higher-order Strategies loading.

### 4.4 Serial indirect association involving interoceptive awareness and emotion regulation difficulties

Analysis of the high-versus-low physical activity contrast indicated distinct indirect statistical associations involving interoceptive awareness and emotion regulation difficulties independently, in addition to a joint indirect association incorporating both variables. However, the primary continuous serial interval did not clearly exclude zero, whereas adjusted, alternative-scoring, and spline BC intervals did. These findings are specification-dependent and provide conditional, rather than uniform, support for an indirect association. Weak measurement indicators further limit interpretation of the intermediary scores. This statistical pattern is compatible with an embodied-cognition framework, which treats bodily awareness, emotion regulation, and behavior as interrelated domains. This aligns with previous research documenting associations between interoceptive awareness and emotion regulation, as well as theoretical accounts positioning the interpretation of bodily signals as foundational to emotional awareness and regulation (Füstös et al., 2013; Price and Hooven, 2018). However, it is crucial to emphasize that the order assigned to these variables represents an analytic specification rather than evidence of temporal precedence. Because cross-sectional indirect-effect estimates may differ substantially from those derived from longitudinal data (O’Laughlin et al., 2018), alternative specifications—including reciprocal and reverse models—remain equally plausible. For instance, emotion regulation difficulties might covary with both physical activity and the appraisal of bodily sensations, while internet addiction could independently correlate with all three variables. Furthermore, the observed associations may stem from unmeasured confounding factors, such as time use, sleep, motivation, psychological distress, or deficits in self-control. Thus, the contribution of the present analysis lies in the simultaneous examination of two statistical intermediaries within a theory-informed model, rather than the verification of a causal chain. The practical implications should be interpreted with the same caution. The findings identify testable targets for future research, but they do not demonstrate that increasing physical activity or interoceptive awareness, or reducing emotion regulation difficulties, would reduce internet addiction. If the proposed ordering is supported in longitudinal and experimental studies, universities could then evaluate programs that combine physical activity with interoceptive-awareness and emotion-regulation components. Such programs require direct testing before recommendations about their effectiveness can be made.

### 4.5 Limitations and future directions

The current study has several limitations should be considered. First, the cross-sectional design precludes causal and temporal inference; therefore, the serial-model estimates represent statistical indirect associations that require confirmation in longitudinal and experimental studies. Second, reliance on self-report scales for all core variables may introduce recall bias, social desirability effects, and residual common method variance. Although Harman’s test revealed no substantial common method variance, future studies should incorporate objective measures, such as accelerometry and digital logs, to minimize self-report bias and enhance measurement fidelity. Third, the online sample from Sichuan Province may limit generalizability. Future research should include more diverse samples and examine relevant contextual factors and dimension-specific patterns of interoceptive awareness. Although sensitivity analyses adjusted for gender, age group, and daily internet-use time, residual confounding from unmeasured variables remains possible. Fourth, IPAQ categorization loses information, and the indirect-association pattern was sensitive to PA operationalization, scoring, and model specification. Fifth, weak IAw and ERD indicators and uneven subscale reliability limit the composite scores; good aggregate CFA fit and item-level alpha do not validate a general factor or a causal intermediary mechanism. Retaining all dimensions preserves content coverage but does not resolve these weaknesses, and the alternative scores were *post hoc* sensitivity analyses. Within these boundaries, our findings provide theory-informed observational evidence; however, subsequent longitudinal and experimental research is ultimately required to determine temporal ordering and confirm causality among these variables.

## 5 Conclusion

In this cross-sectional study of college students, physical activity was negatively associated with internet addiction, with consistent inverse total associations for continuous PA and the high-versus-low contrast. Indirect associations involving interoceptive awareness and emotion regulation difficulties were specification-dependent, and the unadjusted continuous serial interval did not clearly exclude zero. Weak measurement indicators further limit interpretation of the intermediary scores. The cross-sectional design precludes determination of temporal ordering, and reverse directionality remains plausible; for example, lower levels of internet addiction may be associated with higher levels of physical activity. Although the observed pattern was consistent with embodied cognition theory, the study did not establish a causal pathway from physical activity to internet addiction through interoceptive awareness and emotion regulation difficulties. The proposed neural or biological mechanisms were not directly measured and should therefore be regarded only as theoretical possibilities. Accordingly, the findings should be interpreted as observational and hypothesis-generating and do not demonstrate that interventions designed to increase physical activity or interoceptive awareness, or to reduce emotion regulation difficulties, would reduce internet addiction. Future longitudinal and intervention studies are needed to clarify temporal and causal relationships, prospectively test the proposed serial mediation model, and directly assess the proposed neural or biological mechanisms.

## Data Availability

The raw data supporting the conclusions of this article will be made available by the authors, without undue reservation.
