# Supplementary material for: The association between physical activity and internet addiction in college students: a serial mediation analysis of interoceptive awareness and emotion regulation difficulties
Source: Front Psychol. 2026 Oct 6;17:1888809. doi: 10.3389/fpsyg.2026.1888809 (PMC13680448; doi:10.3389/fpsyg.2026.1888809)
Supplement: Supplementary file 1 [file Data_Sheet_1.pdf]

Supplementary analyses of physical activity and measurement quality

S1 Physical activity operationalization and model diagnostics

IPAQ categories were retained because they follow externally specified combinations of frequency, duration and intensity rather than sample-dependent cut points. Moderate and high activity were represented by separate indicators with low activity as the reference; no equally spaced 1-2-3 trend was assumed. Because categorization loses quantitative information, continuous total MET-min/week, expressed per 1,000 units, and a restricted cubic spline sensitivity specification were also analyzed.

Table S1 Distribution of total MET within IPAQ categories.

Group	n	Mean	SD	Median	IQR	Range
All	677	3649.800	2049.227	3504.000	[2010.0, 5172.0]	[66.0, 7875.0]
Low	99	889.136	732.871	577.500	[396.0, 1256.7]	[66.0, 3132.5]
Moderate	195	2286.953	926.008	2225.000	[1637.2, 2772.0]	[495.0, 5256.0]
High	383	5057.269	1395.918	4940.000	[4050.0, 6213.0]	[1539.0, 7875.0]

Note. Overall skewness = 0.152 and excess kurtosis = −0.909. Category ranges overlap because frequency and intensity contribute to classification. IPAQ categories are not simple bins of total MET.

Table S2 Sensitivity analysis comparing categorical and continuous operationalizations of physical activity.

Model	Contrast	B	95% HC3 CI	p	R^2
Unadjusted	Moderate-low	−2.441	[−5.211, 0.329]	0.084	0.063
Unadjusted	High-low	−7.465	[−10.080, −4.851]	< .001	0.063
Unadjusted	Per 1000 MET	−2.031	[−2.431, −1.632]	< .001	0.122
Adjusted	Moderate-low	−2.584	[−5.420, 0.253]	0.074	0.088
Adjusted	High-low	−7.229	[−9.897, −4.562]	< .001	0.088
Adjusted	Per 1000 MET	−1.963	[−2.369, −1.556]	< .001	0.142

Note. n = 677. B is expressed in IA points. Adjusted models include categorical indicators for sex, age group and daily internet-use time. The continuous and category coefficients have different units. The high-low contrast and continuous coefficient were negative; the moderate-low interval included zero.

Table S3 Diagnostics for the continuous serial specification.

Equation	R^2	BP p	Max VIF	Max Cook D	Nonlinear p
IAw	0.291	< .001	1.000	0.027	0.523
ERD	0.200	0.004	1.410	0.004	0.147
IA	0.129	0.762	1.542	0.028	0.002

Note. BP = Breusch–Pagan test. Nonlinear p tests one additional PA spline basis term in that equation. HC3 addresses heteroscedastic standard errors; it does not correct an incorrect mean function. The full spline sensitivity model addresses the observed PA nonlinearity. Residual plots are in Figure S2.

S2 Robust group-comparison analyses

Table S4 ANOVA assumption checks, omnibus group comparisons, and regression diagnostics

Panel A. ANOVA assumptions and group comparisons

Outcome	Levene F(2, 674)	p	Primary omnibus result	η²	follow-up
IAw	32.365	< .001	Welch F(2, 302.563) = 121.789, p < .001	.241	Games-Howell
ERD	1.824	.162	F(2, 674) = 54.655, p < .001	.140	Games-Howell
IA	1.280	.279	F(2, 674) = 22.726, p < .001	.063	Games-Howell

Panel B. Regression diagnostics

Outcome equation	R²	Adjusted R²	Max. VIF	Standardized residual range
IAw	.241	.239	2.114	[-2.731, 3.247]
ERD	.140	.137	2.114	[-2.201, 3.048]
IA	.063	.060	2.114	[-2.307, 3.095]

Note. Levene's tests were based on deviations from group means. Welch's ANOVA was used for interoceptive awareness because homogeneity of variance was violated; conventional one-way ANOVAs were used for the other outcomes. Games–Howell tests were used for pairwise comparisons (Table S2). η^2 values were derived from conventional ANOVA sums of squares and are descriptive for interoceptive awareness. In Panel B, activity level was dummy coded with the low-activity group as the reference. The VIF and residual diagnostics indicated no problematic multicollinearity or marked violations of model assumptions.

Table S5 Games–Howell pairwise comparisons across physical-activity levels

Outcome	Contrast (I − J)	Mean difference	SE	p	95% CI
IAw	Moderate − low	0.069	0.028	.041	[0.002, 0.136]
	High − low	0.398	0.029	< .001	[0.329, 0.466]
	High − moderate	0.329	0.025	< .001	[0.270, 0.388]
ERD	Moderate − low	−0.994	0.926	.531	[−3.179, 1.192]
	High − low	−7.116	0.855	< .001	[−9.137, −5.095]
	High − moderate	−6.122	0.697	< .001	[−7.761, −4.484]
IA	Moderate − low	−2.441	1.405	.194	[−5.760, 0.880]
	High − low	−7.465	1.326	< .001	[−10.600, −4.330]
	High − moderate	−5.024	0.979	< .001	[−7.330, −2.720]

Note. Mean differences are calculated as \(I-J\). Pairwise comparisons were conducted using the Games–Howell procedure. Values are rounded to three decimal places. p values reported as .000 by SPSS are presented as p < .001.

S3 Measurement quality and alternative score specifications

Table S6 Standardized loadings in the aggregate three factor CFA.

Construct	Indicator	Standardized loading	Robust p
IAw	Noticing	0.140	—
	Not-Distracting	0.142	0.063
	Not-Worrying	0.186	0.029
	Attention Regulation	0.502	0.007
	Emotional Awareness	0.389	0.008
	Self-Regulation	0.638	0.008
	Body Listening	0.445	0.009
	Trusting	0.447	0.008
ERD	Nonacceptance	0.301	—
	Goals	0.768	< .001
	Impulse	0.828	< .001
	Awareness	0.086	0.031
	Strategies	0.025	0.532
	Clarity	0.690	< .001
IA	Parcel 1	0.820	—
	Parcel 2	0.824	< .001
	Parcel 3	0.838	< .001
	Parcel 4	0.840	< .001

Note. n = 677. The three-factor CFA was estimated using maximum likelihood with robust standard errors (MLR) and marker-loading identification. Standardized loadings are STDYX estimates. Robust p values refer to the corresponding freely estimated unstandardized loadings; fixed marker loadings have no p value. Model fit was $\chi^2(132) = 204.559$, $p < .001$, CFI = .972, TLI = .967, RMSEA = .028, 90% CI [.021, .036], and SRMR = .042. Despite good overall fit, IAw Noticing, Not-Distracting and Not-Worrying loadings and the ERD Awareness and Strategies loadings were weak; therefore, global fit should not be interpreted as evidence that the respective total scores are unidimensional.

Table S7 Internal consistency of item-level scores.

Score	Items	α	95% bootstrap CI	Domain-rest r
IAw 37 total	37	0.802	[0.779, 0.822]	—
IAw 31 sensitivity	31	0.806	[0.782, 0.826]	—
IAw Noticing	4	0.634	[0.555, 0.696]	0.091
IAw Not-Distracting	6	0.751	[0.708, 0.785]	0.076
IAw Not-Worrying	5	0.659	[0.616, 0.698]	0.080
IAw Attention Regulation	7	0.839	[0.805, 0.866]	0.346
IAw Emotional Awareness	5	0.815	[0.790, 0.836]	0.268
IAw Self-Regulation	4	0.808	[0.776, 0.834]	0.444
IAw Body Listening	3	0.790	[0.742, 0.828]	0.326
IAw Trusting	3	0.825	[0.793, 0.853]	0.304
ERD 36 total	36	0.827	[0.810, 0.842]	—

Score	Items	α	95% bootstrap CI	Domain-rest r
ERD 30 no Awareness	30	0.851	[0.836, 0.865]	—
ERD Nonacceptance	6	0.395	[0.308, 0.470]	0.283
ERD Goals	5	0.833	[0.805, 0.857]	0.570
ERD Impulse	6	0.868	[0.847, 0.887]	0.563
ERD Awareness	6	0.571	[0.509, 0.623]	0.029
ERD Strategies	8	0.755	[0.721, 0.784]	0.035
ERD Clarity	5	0.844	[0.815, 0.868]	0.480
IA 20 total	20	0.896	[0.885, 0.907]	—

Note. $n = 677$, alpha intervals use 5,000 paired participant resamples and the percentile method . Domain-rest r correlates each subscale mean with the remaining item sum. IAw 37-item alpha was .802 and the 31-item sensitivity alpha was .806; deleting six items produced only a very small increase. ERD alpha was .827 and IA alpha was .896. Alpha reflects average item covariance and does not establish unidimensionality or construct validity.

Table S8 Higher-order CFA sensitivity analyses of the Difficulties in Emotion Regulation Scale.

Panel A. Overall fit of higher-order ERD models.

Model	Items / first-order factors	χ^2	df	p	CFI	TLI	RMSEA	90% CI for RMSEA	SRMR
ERD 6 higher order	36 items / 6 domains	1591.437	588	< .001	0.955	0.952	0.050	[0.047, 0.053]	0.054
ERD 5 higher order	30 items / 5 domains	1221.400	400	< .001	0.962	0.958	0.055	[0.052, 0.059]	0.056

Panel B. Standardized first-order domain loadings on the higher-order ERD factor.

Model	First-order domain	Standardized loading, λ	SE	P	90% CI
36-item / 6-domain	Nonacceptance	0.444	0.054	—	[0.356, 0.533]
	Goals	0.841	0.018	0.004	[0.811, 0.871]
	Impulse	0.897	0.016	0.005	[0.871, 0.923]
	Awareness	0.138	0.035	0.024	[0.080, 0.196]
	Strategies	0.011	0.043	0.799	[−0.060, 0.082]
	Clarity	0.765	0.019	0.005	[0.734, 0.796]
30-item / 5-domain	Nonacceptance	0.448	0.054	—	[0.359, 0.537]
	Goals	0.840	0.018	0.005	[0.810, 0.870]
	Impulse	0.897	0.016	0.005	[0.870, 0.923]
	Strategies	0.012	0.043	0.771	[−0.058, 0.083]
	Clarity	0.766	0.019	0.005	[0.735, 0.798]

Note. The 6-domain model used 36 items representing Nonacceptance, Goals, Impulse, Awareness, Strategies, and Clarity. The 5-domain sensitivity model omitted the six Awareness items and retained the remaining 30 items. Items were treated as ordered categorical indicators and estimated using WLSMV. Standardized loadings and standard errors are STDYX estimates. p values refer to the corresponding freely estimated unstandardized higher-order loadings. Higher scores indicate greater emotion-regulation difficulties. The unstandardized loading of Nonacceptance on the higher-order ERD factor was fixed to 1 for model identification; therefore, no significance test is reported for that parameter, although its standardized loading is reported. The two models should be interpreted as sensitivity specifications rather than as nested models compared by the ordinary chi-square difference test.

S4 Mediation model, decomposition, and robustness analyses

The four-variable serial mediation model was retained as the primary analysis. A sensitivity model additionally included gender (female vs. male), age group, and daily internet-use time (M = 2.89, SD = 1.065) as covariates of interoceptive awareness, emotion regulation difficulties, and internet addiction. Models were estimated using maximum likelihood with 5,000 bias-corrected bootstrap draws (N = 677). PA2 and PA3 denote moderate and high physical activity, respectively, relative to low physical activity. Because the path models were saturated, global fit indices are not informative and are not reported.

Table S9 Parameter estimates from the covariate-adjusted sensitivity model.

Outcome and predictor	b (SE)	β (SE)	p	95% BC bootstrap CI for β
IAw				
PA2 (moderate vs. low)	0.071 (0.028)	0.090 (0.036)	.011	[0.020, 0.159]
PA3 (high vs. low)	0.393 (0.029)	0.544 (0.035)	< .001	[0.473, 0.611]
Gender (female vs. male)	-0.065 (0.024)	-0.090 (0.033)	.008	[-0.153, -0.023]
Age group	0.050 (0.024)	0.071 (0.034)	.038	[0.002, 0.138]
Daily internet-use time	-0.001 (0.012)	-0.004 (0.035)	.901	[-0.072, 0.067]
ERD				
IAw	-5.265 (0.971)	-0.219 (0.040)	< .001	[-0.298, -0.138]
PA2 (moderate vs. low)	-0.645 (0.901)	-0.034 (0.047)	.474	[-0.124, 0.062]
PA3 (high vs. low)	-4.952 (0.945)	-0.285 (0.054)	< .001	[-0.389, -0.175]
Gender (female vs. male)	-0.091 (0.622)	-0.005 (0.036)	.884	[-0.075, 0.067]
Age group	-0.865 (0.605)	-0.051 (0.036)	.153	[-0.119, 0.021]
Daily internet-use time	0.107 (0.282)	0.013 (0.035)	.706	[-0.057, 0.082]
IA				
ERD	0.171 (0.059)	0.123 (0.043)	.004	[0.034, 0.205]
IAw	-3.563 (1.434)	-0.107 (0.043)	.013	[-0.190, -0.021]
PA2 (moderate vs. low)	-2.011 (1.389)	-0.076 (0.053)	.148	[-0.178, 0.029]
PA3 (high vs. low)	-4.850 (1.526)	-0.202 (0.063)	.001	[-0.323, -0.076]
Gender (female vs. male)	1.219 (0.906)	0.051 (0.038)	.179	[-0.023, 0.127]
Age group	0.653 (0.844)	0.028 (0.036)	.439	[-0.043, 0.098]
Daily internet-use time	-0.403 (0.430)	-0.036 (0.038)	.348	[-0.112, 0.039]

Note. b = unstandardized estimate; β = STDYX standardized estimate; BC = bias-corrected. Two-tailed p values are from the model output. R^2 = .255 for interoceptive awareness, .180 for emotion regulation difficulties, and .094 for internet addiction.

Table S10 Regression paths in the continuous physical-activity serial mediation model.

Outcome (R^2)	Predictor	*b*	SE	β	*p*	95% CI for b
**IAw (R^2 = .291)**	Physical activity, per 1,000 MET-min/week	0.094	0.006	0.539	< .001	[0.082, 0.106]
**ERD (R^2 = .200)**	Physical activity, per 1,000 MET-min/week	−1.370	0.178	−0.326	< .001	[−1.713, −1.014]
	IAw	−4.262	0.969	−0.177	< .001	[−6.200, −2.359]
**IA (R^2 = .129)**	Physical activity, per 1,000 MET-min/week	−1.715	0.275	−0.295	< .001	[−2.240, −1.166]
	IAw	−1.353	1.467	−0.041	.357	[−4.154, 1.588]

Outcome (R^2)	Predictor	*b*	SE	β	*p*	95% CI for b
	ERD	0.107	0.058	0.077	.067	[−0.010, 0.217]

Note. b = unstandardized coefficient; SE = standard error; β = standardized coefficient;BC CI = bias-corrected bootstrap confidence interval based on 5,000 resamples. All p values are two-tailed. Physical activity was expressed per 1,000 MET-min/week. Because the model was saturated, R^2 and individual paths, rather than global fit indices, were used to summarize the model.

Table S11 Full decomposition using rebuilt 37-item IAw and 36-item ERD scores.

Contrast	Effect	B	95% BC CI	95% percentile CI
Moderate-low	Direct	−2.021	[−4.756, 0.781]	[−4.836, 0.694]
Moderate-low	Via IAw	−0.253	[−0.648, −0.036]	[−0.593, −0.016]
Moderate-low	Via ERD	−0.105	[−0.497, 0.179]	[−0.462, 0.207]
Moderate-low	Serial	−0.062	[−0.168, −0.010]	[−0.151, −0.006]
Moderate-low	Total indirect	−0.420	[−0.959, −0.011]	[−0.934, 0.005]
Moderate-low	Total	−2.441	[−5.146, 0.422]	[−5.270, 0.303]
High-low	Direct	−4.810	[−7.872, −1.813]	[−7.780, −1.735]
High-low	Via IAw	−1.462	[−2.593, −0.331]	[−2.590, −0.324]
High-low	Via ERD	−0.835	[−1.635, −0.261]	[−1.581, −0.230]
High-low	Serial	−0.358	[−0.707, −0.113]	[−0.682, −0.096]
High-low	Total indirect	−2.655	[−4.016, −1.329]	[−4.022, −1.332]
High-low	Total	−7.465	[−10.090, −4.836]	[−10.112, −4.875]
Per 1000 MET	Direct	−1.715	[−2.240, −1.166]	[−2.250, −1.171]
Per 1000 MET	Via IAw	−0.127	[−0.396, 0.149]	[−0.395, 0.151]
Per 1000 MET	Via ERD	−0.146	[−0.328, 0.011]	[−0.331, 0.010]
Per 1000 MET	Serial	−0.043	[−0.103, 0.000]	[−0.097, 0.003]
Per 1000 MET	Total indirect	−0.317	[−0.658, 0.018]	[−0.658, 0.018]
Per 1000 MET	Total	−2.031	[−2.428, −1.637]	[−2.433, −1.643]

Note. Note. n = 677. Indirect effects were estimated using 5,000 bootstrap resamples. BC intervals are bias-corrected bootstrap confidence intervals, whereas percentile intervals are percentile bootstrap confidence intervals. In the continuous specification (per 1,000 MET), the IAw-only and ERD-only indirect effects included zero under both interval conventions; the serial indirect effect reached zero at the reported precision under the BC interval and included zero under the percentile interval. In the categorical specification, the serial indirect effects for both moderate-versus-low and high-versus-low contrasts excluded zero under both interval conventions. The high-versus-low total indirect effect also excluded zero under both interval conventions, with the IAw-only pathway showing the largest specific indirect estimate. For the moderate-versus-low contrast, the total indirect effect excluded zero under the BC interval but narrowly included zero under the percentile interval. All indirect effects should be interpreted as exploratory associations because the temporal ordering of the proposed mediators and outcome cannot be established from these data.

Table S12 Robustness of the serial indirect association across alternative model specifications.

Panel A. Physical-activity operationalization and covariate adjustment.

Specification	PA coding	Contrast	Serial indirect B	95% BC CI	Bootstrap inference
Primary corrected-score model	categorical	Moderate-low	−0.062	[−0.168, −0.010]	Supported
		High-low	−0.358	[−0.707, −0.113]	Supported

Specification	PA coding	Contrast	Serial indirect B	95% BC CI	Bootstrap inference
Primary corrected-score model	continuous	Per 1,000 MET-min/week	−0.043	[−0.103, 0.000]	Not clearly supported / borderline
Covariate-adjusted model	categorical	Moderate-low	−0.064	[−0.172, −0.012]	Supported
		High-low	−0.353	[−0.703, −0.116]	Supported
Covariate-adjusted model	continuous	Per 1,000 MET-min/week	−0.043	[−0.102, −0.003]	Supported

Panel B. Alternative mediator-score specifications.

Specification	PA coding	Contrast	Serial indirect B	95% BC CI	Bootstrap inference
IAw-31 sensitivity	categorical	Moderate-low	−0.058	[−0.163, −0.013]	Supported
		High-low	−0.329	[−0.651, −0.109]	Supported
	continuous	Per 1,000 MET-min/week	−0.038	[−0.094, −0.003]	Supported
ERD-30 sensitivity	categorical	Moderate-low	−0.053	[−0.148, −0.009]	Supported
		High-low	−0.304	[−0.607, −0.082]	Supported
	continuous	Per 1,000 MET-min/week	−0.048	[−0.106, −0.006]	Supported

Panel C. Nonlinear physical-activity specification.

Specification	Contrast	Serial indirect B	95% BC CI	Bootstrap inference
Restricted cubic spline	P75 vs P25 of total MET-min/week	−0.148	[−0.340, −0.014]	Supported

Note. B denotes the unstandardized serial indirect association through interoceptive awareness and emotion regulation difficulties in sequence. BC CI = bias-corrected bootstrap confidence interval based on 5,000 resamples. Categorical models used low physical activity as the reference group. Continuous linear models expressed physical activity per 1,000 MET-min/week. The restricted cubic spline specification represents the serial indirect association comparing the 75th with the 25th percentile of total MET-min/week and is therefore not directly comparable in magnitude with the categorical or linear continuous coefficients. IAw-31 and ERD-30 were post hoc alternative scoring specifications used for sensitivity analyses and were not treated as replacements for the prespecified primary scores.

Supplementary figures

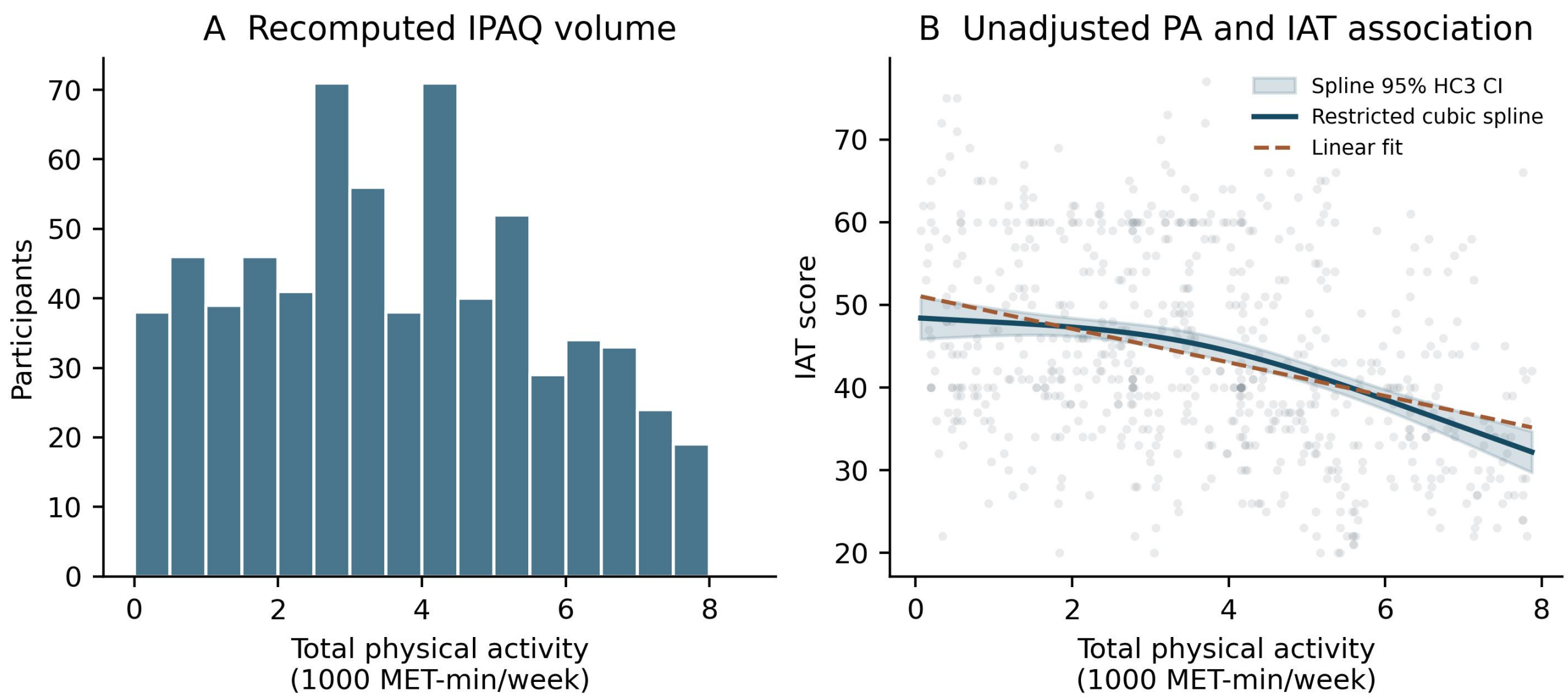

Figure S1 Physical activity distribution and association with IA.

Note. n = 677. The spline has knots at the 10th, 50th and 90th PA percentiles. The shading is a pointwise 95% HC3 confidence interval for the fitted mean. The nonlinear term has p = .003. The figure does not demonstrate a causal dose response or an activity threshold.

Residual diagnostics

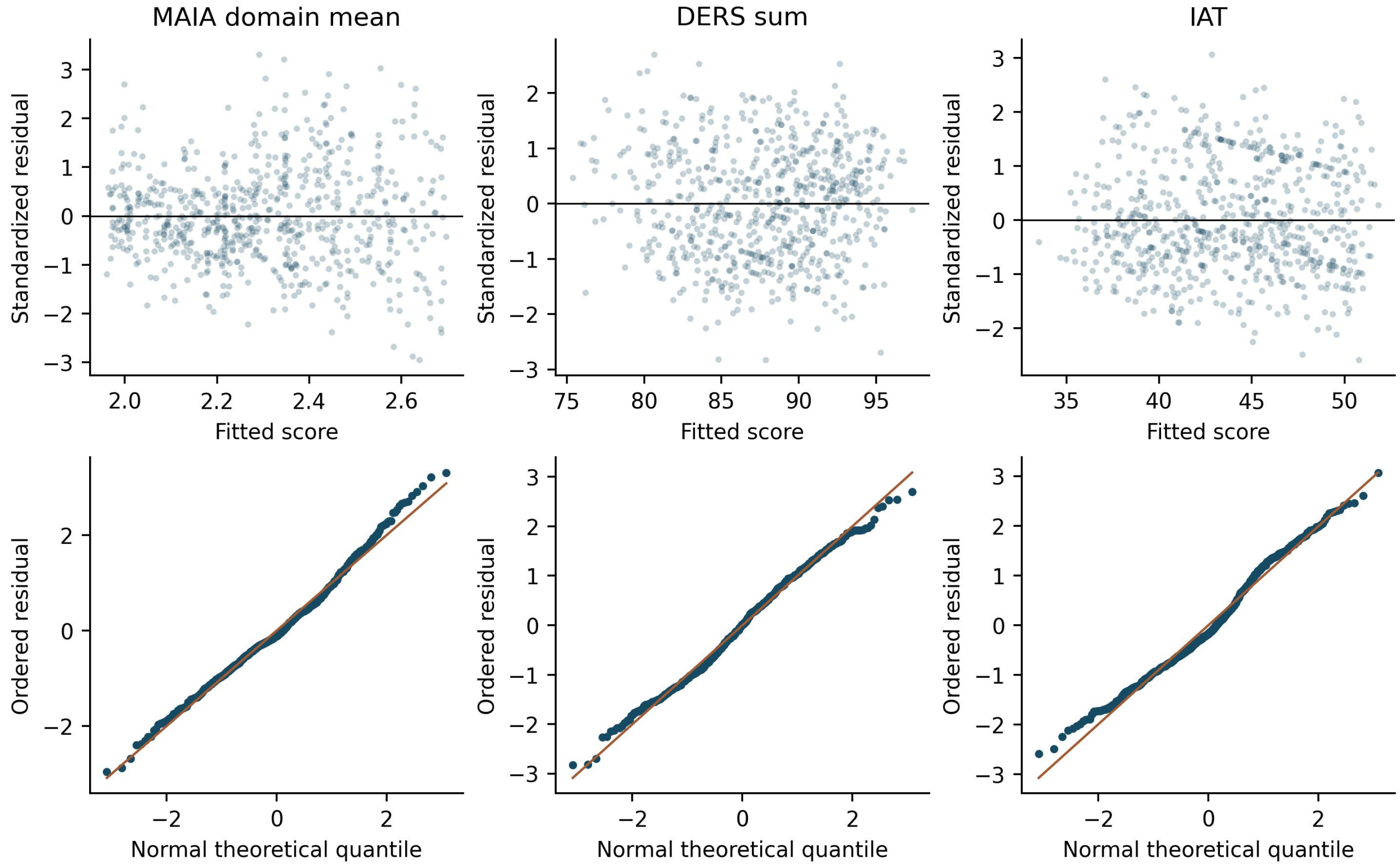

Figure S2 Residual diagnostics for the continuous model using the IAw domain-mean score.

Note. Top: standardized residuals versus fitted values. Bottom: normal quantile plots. Columns represent IAw, ERD and IA equations, respectively. These diagnostics address regression behavior, not measurement validity.

Figure S3. Comparison of standardized focal estimates in the primary four-variable model and the covariate-adjusted sensitivity model.

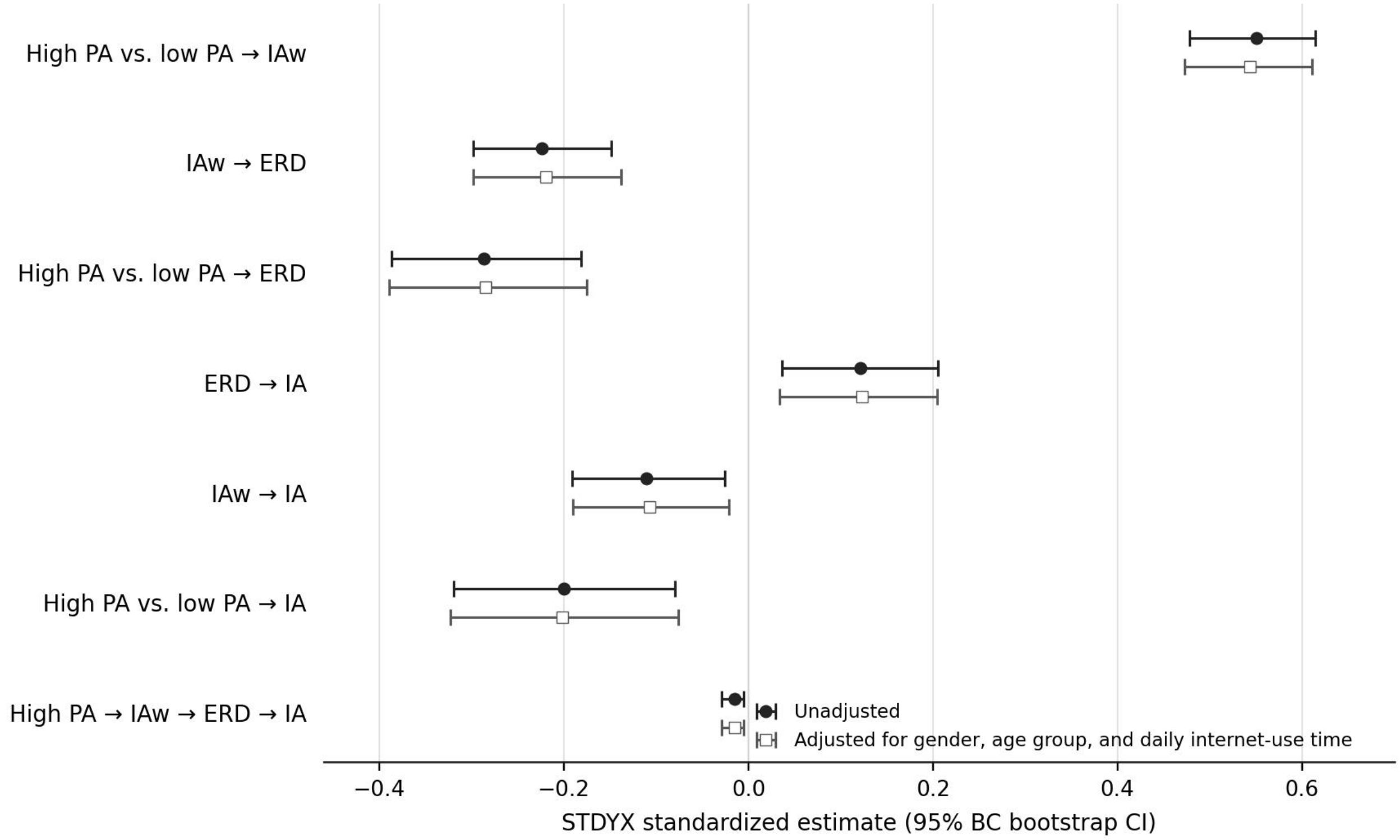

Note. Points denote STDYX standardized estimates and horizontal bars denote 95% bias-corrected bootstrap confidence intervals. The serial indirect association is shown on the same scale as the direct path estimates to demonstrate its stability across model specifications
